# Effect of nurse-performed enhanced patient education on colonoscopy bowel preparation quality

**DOI:** 10.1590/1518-8345.5597.3627

**Published:** 2022-08-12

**Authors:** Gamze Arslanca, Mahmure Aygün

**Affiliations:** 1 Prof. Dr. Cemil Taşcıoğlu City Hospital, Colonoscopy Department, Istanbul, Turquia.; 2 Biruni University, Graduate Education Institute Nursing Program, Istanbul, Turquia.

**Keywords:** Colonoscopy, Bowel Preparation, Nurse-Performed Cecal Intubation, Patient Education, Quality Indicators, Colonoscopia, Preparação Intestinal, Intubação Cecal Realizada por Enfermeiros, Educação do Paciente, Indicadores de Qualidade, Colonoscopia, Preparación Intestinal, Intubación Cecal Realizada por Enfermeros, Educación del Paciente, Indicadores de Calidad

## Abstract

**Objective:**

to evaluate the effect of nurse-performed enhanced patient education sessions on adequate bowel preparation and cecal intubation rates.

**Method:**

a prospective, quasi-experimental, comparative study with a quantitative approach. The intervention group (n=150) received education enhanced by a visual presentation and reminder calls. The control group (n=156) received the clinic’s standard written instructions. Adequate bowel preparation rates and other colonoscopy quality indicators were compared between the groups.

**Results:**

Boston Bowel Preparation scale scores and adequate bowel preparation rates were higher in the intervention group than in the control group (respectively, 6.76±2.1 *vs.* 5.56±2.4, p=0.000, and 80% *vs.* 69.2%, p=0.031). The cecal intubation rates were higher in the intervention group (80% *vs.* 67.3%, p=0.012). Due to inadequate bowel preparation, unsuccessful cecal intubation rates were 0% in the intervention group and 17.6% in the control group. Biopsy rates were higher in the intervention group (28% *vs.* 13.3%, p=0.002).

**Conclusion:**

the nurse-performed enhanced patient education sessions increase adequate bowel preparation rates and, in parallel, cecal intubation rates. To reach the colonoscopy quality standards recommended in the guidelines, it is suggested that patient education be supported by different training tools and given by health professionals.

## Resumo

**Objetivo:**

avaliar o efeito da educação aprimorada realizada por enfermeiros no preparo intestinal adequado e na taxa de intubação cecal.

**Método:**

estudo prospetivo, *quasi*-experimental e comparativo com abordagem quantitativa. Os pacientes no grupo de intervenção (n=150) receberam a educação aprimorada e telefonemas para lembrá-los do procedimento. O grupo de controle (n=156) recebeu as instruções padrão da clínica por escrito. As taxas de preparo intestinal adequado e outros indicadores da qualidade da colonoscopia foram comparadas entre os grupos.

**Resultados:**

as pontuações obtidas na *Boston Bowel Preparation Scale* e a taxa de preparo intestinal adequado foram maiores no grupo de intervenção do que no grupo de controle (6,76±2,1 *vs.* 5,56±2,4, p=0,000, e 80% *vs.* 69,2%, p=0,031). A taxa de intubação cecal foi mais alta no grupo de intervenção (80% *vs.* 67,3%, p=0,012). A taxa de intubação cecal malsucedida devido ao preparo intestinal inadequado foi de 0% no GI, enquanto que o GC obteve uma taxa de 17,6%. Além disso, a taxa de biópsia foi maior no grupo de intervenção (28% *vs.* 13,3%, p=0,002).

**Conclusão:**

a educação do paciente aprimorada e realizada por enfermeiros aumenta as taxas de preparo intestinal adequado e, consequentemente, as taxas de intubação cecal. Para alcançar os padrões de qualidade de colonoscopia recomendados nas diretrizes, recomendamos que a educação do paciente inclua diferentes ferramentas de treinamento e seja realizada por profissionais da saúde.

## Resumen

**Objetivo:**

evaluar el efecto de la educación perfeccionada realizada por enfermeros en la preparación intestinal adecuada y en las tasas de intubación cecal.

**Método:**

estudio prospectivo, casi experimental y comparativo, con abordaje cuantitativo. El grupo de intervención (n=150) recibió la educación perfeccionada y llamadas telefónicas recordatorias. El grupo de control (n=156) recibió las instrucciones estándar de la clínica por escrito. Las tasas de preparación intestinal adecuadas y otros indicadores de la calidad de la colonoscopia fueron comparadas entre los grupos.

**Resultados:**

las puntuaciones obtenidas en la *Boston Bowel Preparation Scale* y las tasas de preparación intestinal adecuadas fueron en el grupo de intervención mayores que en el grupo de control (6,76±2,1 *vs.* 5,56±2,4, p=0,000, y 80% *vs.* 69,2%, p=0,031, respectivamente). Las tasas de intubación cecal fueron mayores en el grupo de intervención (80% *vs.* 67,3%, p=0,012). La tasa de intubación cecal fracasada debido a la preparación intestinal inadecuada fue de 0% en el GI, en cuanto que el GC obtuvo una tasa de 17,6%. Además de eso, las tasas de biopsia fueron mayores en el grupo de intervención (28% *vs.* 13,3%, p=0,002).

**Conclusión:**

la educación del paciente perfeccionada y realizada por enfermeros aumenta las tasas de preparación intestinal adecuadas y, consecuentemente, las tasas de intubación cecal. Para alcanzar los estándares de calidad de colonoscopia recomendados en las directrices, recomendamos que la educación del paciente sea apoyada por diferentes herramientas de entrenamiento y realizada por profesionales de la salud.

**Highlights**

(1) The BBPS scores were higher in the enhanced patient education group (IG) (6.76 *vs.* 5.56).

(2) Adequate bowel preparation (BP) was higher in IG (80% *vs.* 69.2%).

(3) Cecal intubation rates were higher in IG (80% *vs.* 69.2%).

(4) Biopsy rates were higher in IG (28% *vs.* 13.3%).

(5) Cecal intubation failure due to inadequate BP was higher in CG (17.6% *vs.* 0%).

## Introduction

Colonoscopy is a lower gastrointestinal endoscopy performed to screen, diagnose, and treat colon and terminal ileum pathologies. Colonoscopy significantly reduces the incidence and mortality of colorectal cancer (CRC), allowing the detection and removal of precancerous lesions and early-stage CRC^1^ ^-^ ^3^.

The most critical performance indicators for colonoscopy are rates of adequate bowel preparation (BP), cecal intubation (CI), and adenoma detection (AD). Adequate BP refers to bowel cleansing, which will ensure proper visualization of the colonic mucosa. Inadequate BP increases risk, such as missed pathological lesions and repeats colonoscopy^4^ ^-^ ^8^.

There are many clinician and patient-related factors that affect the adequate BP process. These are types of used bowel-cleansing agents and/or purgatives; patients’ compliance with BP medications, dietary restrictions, additional fluid intake; waiting time for the procedure^2^ ^,^ ^5^ ^-^ ^7^ ^,^ ^9^. 

Patients have a central role in the BP process because the BP process involves instructions that patients will follow. It is very difficult to manage the process, especially for patients who will undergo a first-time colonoscopy. Patients’ compliance with these instructions exactly is an essential factor for adequate BP. Inadequate BP is the primary cause of incomplete colonoscopies in clinical^2^ ^,^ ^10^. Reasons such as insufficient explanations of the meaning and importance of adequate bowel cleansing or patients’ not understanding the instructions well/forgetting the timing of the instructions cause inadequate bowel cleansing and unsuccessful colonoscopy. Therefore, the guidelines place a particular emphasis on the use of enhanced patient education for adequate BP*.* It is recommended that enhanced education should be provided by health care professionals and combine written and verbal instructions^2^ ^,^ ^5^ ^-^ ^7^ *.* Recent studies provide strong evidence that enhanced patient education provides better bowel cleansing and patient compliance than standard instructions^11^ ^-^ ^17^.

In this study, we primarily aimed to investigate the effect of nurse-performed enhanced patient education on adequate bowel preparation in patients who were given face-to-face education supported by a visual presentation and who received reminder phone calls before the procedure. In addition, patients’ thoughts on their experience with the procedure will be evaluated. The study’s secondary aim was to evaluate the effect of enhanced patient education on colonoscopy performance measures (e.g. cecal intubation rates, cecal intubation times, withdrawal times, polyp detection rates). 

## Method

This prospective, quasi-experimental, comparative, single-center, endoscopist-blinded study was carried out in the colonoscopy unit of an educational research hospital in Istanbul, between July and December 2018. 

The study’s primary aim was to evaluate the effect of the enhanced patient education on adequate BP and, secondarily, on other colonoscopy performance criteria. 

### Study population

**Figure 1:**
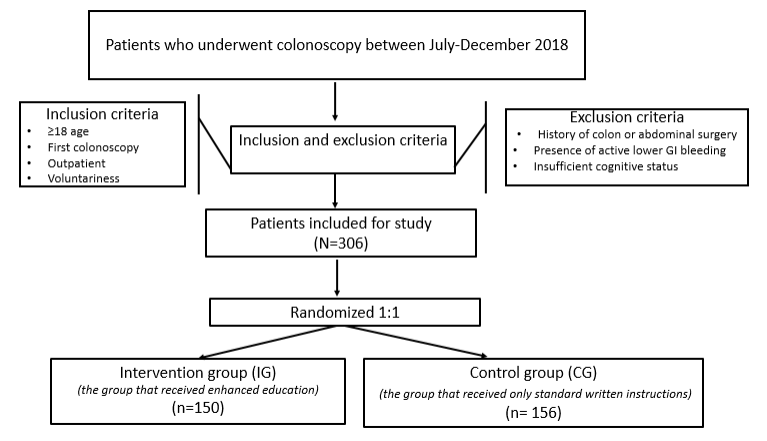
Study flowchart

The inclusion criteria were (1) patients from the outpatient clinic, (2) aged 18 and over, (3) having a first-time colonoscopy, and (4) voluntariness to participate in the study. Patients who had undergone abdominal surgery before, patients with active lower gastrointestinal bleeding, and patients with any cognitive impairment like dementia in their medical history were excluded from the study (Figure 1).

Indications for colonoscopy were routine screening, positive fecal occult blood test, iron deficiency anemia, changes in stool characteristics, and history of rectal bleeding. Bowel preparation medications were prescribed by endoscopists who were blinded to groups’ information. 

One of the researchers was working as a full-time colonoscopy nurse in the unit. The patients were getting their colonoscopy appointment from the unit’s medical secretary. The medical secretary was informed about the inclusion and exclusion criteria of the study and divided patients who met the inclusion criteria into intervention (IG) and control (CG) groups, consecutively, according to the order of arrival for an appointment. The patients assigned to the IG were directed to the research nurse. The nurse researcher trained the patients in the IG on the same day. The process of assigning the patients to the groups and the training of the IG patients was repeated every day. This researcher was responsible for the training (face to face and by telephone calls) of the patients in the IG. In addition, this research nurse assisted in the colonoscopy procedure of all patients (IG and CG) and recorded the data of the procedure.

The G* Power 3.1 program was used to calculate the sample size. The sample size was found as a total of 176 people (88 *per* arm), using the difference between two independent means, with 5% significance and 95% power^18^. The study was conducted with a total of 306 patients (intervention= 150, control= 156).

### Data collection

The CG was given a leaflet that consists of one page and is routinely used in the endoscopy unit for BP. The IG was provided with face-to-face education, including a visual presentation by an experienced endoscopy nurse (researcher) who answered all the questions. The researcher carried out the patient education sessions in a room reserved for education, with an average of 10 minutes for each patient. Also, the patients received a booklet containing BP instructions with colorful pictures. Two days before the procedure, patients were called by phone against the possibility of having forgotten the instructions, and their questions were answered. The process of calling the patients in the IG by phone and reminding the instructions was done by the nurse researcher who gave the training.

The visual presentation’s content and design were prepared using examples in the literature and experiences^6^ ^,^ ^19^ ^-^ ^21^. The content of education was included (1) the purpose, importance, and stages of colonoscopy, (2) the purpose and start of purgative-laxatives, (3) the purpose of consuming a clear liquid diet, and additional fluid, (4) the meaning and importance of adequate bowel cleansing, (5) last stool color, (6) side effects of the regimen. Visual presentation and booklet were supported with colorful pictures to help patients visualize optimal and poor bowel cleansing.

Colonoscopy examinations were performed by experienced endoscopists who were blinded to the group information. The data recorded in the study are: The patients’ socio-demographic and clinical characteristics; BP Adequacy [Boston bowel preparation scale (BBPS) score ≥5]; CI (represent the completion of the procedure); Cecal intubation time (CIT) (the time interval between the insertion of scope and arrival at the cecum); withdrawal time (WT) (the time taken to retract of the scope from the cecum into the anus); total processing time (the sum of the CI and WT); polyp detection rate (PDR)^4^ ^-^ ^5^ ^,^ ^8^; the patients’ compliance level with the BP regimen and their thoughts on the experience after the procedure. 

Bowel preparation adequacy was evaluated by the endoscopist using the BBPS. The BBPS is a bowel cleanliness rating scale in which three main colonic regions (the right side, the transverse section, and the left side) are evaluated in the range of 0-3 points (0: unprepared colon segment; 1: major residual stool or opaque liquid; 2: minor residual staining; and 3: entire mucosa of colon segment seen well). The total BBPS score would range from 0 to 9, with a higher score reflecting good-quality bowel cleansing^21^.

Approval was obtained from the Research Ethics Committee of the Biruni University (Decision number: 16-11, Date: 30.05.2018) and the hospital administration (opinion number: 48670771-771). The study was performed in compliance with the ethical principles of the Declaration of Helsinki^22^. Informed consent was obtained from the patients.

### Statistical analysis

Statistical analysis of the data was evaluated using the SPSS Statistics 22.0 program (IBM Corp.). Before assessing the data, the normality of data distribution was evaluated using the Shapiro-Wilks test. Continuous data with normal distributions are expressed as mean and standard deviation, while categorical data are presented as numbers and frequencies. The Kruskal-Wallis test was used for comparisons between more than two non-normally distributed variables. The Mann-Whitney U test was used to compare the difference between two variables that did not conform to normal distribution. The Chi-Square test, Fisher-Freeman-Halton Exact test, Student t-test, and Yates continuity correction test were used for statistical differences. The Pearson correlation analysis was used to test the direction and strength of the relationship between age and BBPS scores. The statistical significance level was determined as 0.05.

## Results

In the study, 306 patients were included; 137 (44.8%) were female, and 169 (55.2%) were male. The average age was 55.2 ± 15, and 168 (54.9%) of the patients had primary education. There was no significant difference between the two groups in terms of these socio-demographic variables (sex: p= 0.339; age: p= 0.213; education: p= 0.303). Body mass index (BMI) in 206 (65.3%) patients was ≥ 25 (Table 1). 

**Table 1:** Socio-demographic and clinical features of patients. Istanbul, Turkey, 2018

Parameters		Total	Intervention group	Control Group	p
		mean ± SD^*^	mean ± SD	mean ± SD	
Age		55.2±15	54.1±15,0	56.3±14,9	0.213^†^
		n(%)	n(%)	n(%)	
Gender	Female	137 (44.8)	63 (42)	74 (47.4)	0.339^‡^
	Male	169 (55.2)	87 (58)	82 (52.6)	
Education	İlliterate	29 (9.5)	12 (8)	17 (10.9)	0.303^‡^
	Primary school	168 (54.9)	81 (54)	87 (55.8)	
	Secondary school	55 (18)	24 (16)	31 (19.9)	
	High school	36 (11.8)	21 (14)	15 (9.6)	
	University	18 (5.9)	12 (8)	6 (3.8)	
Appointment waiting time	< 2 weeks	48 (15.7)	39 (26)	9 (5.8)	0.000^‡^
	3-6 weeks	185 (60.5)	90 (60)	95 (60.9)	
	>7 weeks	73 (23.9)	21 (14)	52 (33.3)	
BMI^¶^	<18.5	3 (1)	0 (0)	3 (1.9)	0.037^§^
	18.5-24.9	97 (31.7)	39 (26)	58 (37.2)	
	25-29.9	164 (53.6)	90 (60)	74 (47.4)	
	30-34.9	42 (3.7)	21 (14)	21 (13.5)	
Chronic diseases	Heart Disease	62 (20.3)	27 (18)	35 (22.4)	0.335^‡^
	Hypertension	113 (36.9)	48 (32)	65 (41.7)	0.080^‡^
	Diabetes Mellitus	48 (15.7)	24 (16)	24 (15.4)	0.882^‡^
	Other chronic diseases	31 (10.1)	15 (10)	16 (10.3)	1.000^‡^

Data are presented as *mean ± standard deviation; ^†^Student’s t-Test; ^‡^Chi-Square Test; ^§^Fisher-Freeman-Halton Test; ^¶^BMI = Body Mass Index

There was no difference between the two groups in terms of family history of GI cancer, positive fecal occult blood and iron deficiency anemia indication, chronic diseases, and the types of medications used continuously (p> 0.05). 

In the IG, more patients waited less than two weeks than in the CG [39 (26%) *vs*. 9 (5.8%), p = 0.000] (Table 1). 255 (83.3%) of the cases had colonoscopy without sedation, and there was no difference between the groups (p = 0.281).

The findings of the study and control groups regarding BP and CI are shown in Table 2. The mean BBPS score of the patients was 6.1 ± 2.3. The total BBPS score was higher in the IG than the CG (6.76 ± 2.1 *vs*. 5.56 ± 2.4, p = 0.000). The BBPS score of those with adequate BP (BBPS ≥5 score) in the IG was significantly higher than the CG (7.6 ± 1.1 *vs.* 6.9 ± 1.4, p = 0.000). Adequate BP rate in the IG was higher than the CG [120 (80%) *vs.* 108 (69.2%), p = 0.031]. The CI rate was significantly higher in the IG compared to the CG [120 (80%) *vs.* 105 (67.3%), p = 0.012]. There was no CI failure due to inadequate bowel cleansing in the IG (Table 2).

**Table 2:** Bowel preparation outcomes. Istanbul, Turkey, 2018

Parameters		Total (N:306)	Intervention Group (n:150)	Control Group (n:156)	p
		mean ± SD^*^	mean ± SD	mean ± SD	
Total BBPS^§^ Score		6.1±2.3	6.7±2.1	5.5±2.4	0.000^†^
Total BBPS^§^ Score	BBPS^§^: ≥ 5	228 (74.5)	7.6±1.1*	6.9±1.4*	0.000^†^
	BBPS^§^: < 5	78 (25.5)	3.1±1.0*	2.5±1.1*	0.297^†^
		n(%)	n(%)	n(%)	
Adequate BP^||^ rates according to the BBPS^§^ score	Adequate: BBPS^§^: ≥ 5	228 (74.5)	120 (80)	108 (69.2)	0.031^‡^
	Inadequate: BBPS^§^: < 5	78 (25.5)	30 (20)	48 (30.8)	
Cecal intubation rates	Yes	225 (73.5)	120 (80)	105 (67.3)	0.012^‡^
	No	81 (26.5)	30 (20)	51 (32.7)	
Reasons for unsuccessful cecal intubation	Patient intolerance	57 (70.4)	21 (70)	36 (70.6)	0.013^‡^
	Inadequate bowel preparation	9 (11.1)	0 (0)	9 (17.6)	
	Occlusive lesion	15 (18.5)	9 (30)	6 (11.8)	

Data are presented as ^*^mean±standard deviation; ^†^Mann-Whitney U test; ^‡^Chi-Square Test; ^§^BBPS = Boston Bowel Preparation Scale, ^||^BP = Bowel preparation

The mean CIT was 8.83 ± 4.3 minutes among the CI patients (N: 225, 73.5%). The WT found was 11.0±6.2 minutes in interventional procedures and 4.3±2.0 minutes in nonintervention procedures. There was no difference between the groups (CIT: p=0.350; WT-interventional: p=0.246; WT-nonintervention: p=0.237). The polyp detection rate was 95 (31.4%) in the whole series, and the biopsy was performed on 62 (20.7%) patients. There was no difference in PDR between the groups (36% *vs.* 26.8%, p = 0.084) (Table 3). 

There was no significant relationship between age and the BBPS scores in the IG (r: -0.030, p = 0.712). A significant positive relationship was found between age and BBPS scores in the CG (r: 0.177, p: 0.027). While there was no difference between the BBPS scores in terms of sex in the IG (p = 0.059), the BBPS score was higher in males in the CG (p = 0.000) (Table 4). When the effect of education level on the BBPS score was examined, no significant difference was found in both groups (p> 0.05). 

**Table 3:** Colonoscopy outcomes. Istanbul, Turkey, 2018

Parameters		Total (N=306)	Intervention Group (n=150)	Control Group (n=156)	p
		mean ± SD^*^	mean ± SD	mean ± SD	
Cecal intubation time (min) *(n=225)*		8.8±4.3	8.9±3.9	8.7±4.7	0.350^†^
Withdrawal time^||^ in intervention procedures (min) *(n=225)*		11.0±6.2	12.3±7,0	9.5±4.8	0.246^†^
Withdrawal time^||^ in nonintervention procedures (min) *(n=73)*		4.3±2.0	4.0±1.2	4.7±2.6	0.237^†^
		n(%)	n(%)	n(%)	
Length of procedure ^#^ (min) *(N=225; study:120, control:105)*	<30 min	205 (91.1)	108 (90)	97 (92.4)	0.696^‡†^
	30-60 min	20 (8.9)	12 (10)	8 (7.6)	
Polyp detection rates *(N=303, study:150, control:153)*	Yes	95 (31.4)	54 (36)	41 (26.8)	0.084^§^
	No	208 (68.6)	96 (64)	112 (73.2)	
Biopsy *(N=300, study:150, control:150)*	Yes	62 (20.7)	42 (28)	20 (13.3)	0.002^§^
	No	238 (79.3)	108 (72)	130 (86.7)	

Data are presented as ^*^mean±standard deviation, ^†^Mann-Whitney U test; ^‡^Yates Continuity Correction test; ^§^Chi-Square Test; Min = Minute, ^||^WT = Withdrawal time

The BBPS scores did not differ between waiting times in the IG (p = 0.270). In the CG, patients who waited for less than or equal to two weeks had a higher BBPS score (p = 0.032). In both groups, low BMI was associated with high BBPS score (IG: p = 0.000; CG: p = 0.005) (Table 4).

**Table 4:** Effect of demographic and clinical data on BBPS score. Istanbul, Turkey, 2018

Parameters		BBPS^§^ scores			
		Intervention Group (n=150)	p	Control Group (n=156)	p
		mean ± SD*		mean ± SD	
Gender	Female	6.9±2.3	^ⱡ^0.059	4.6±2.4	0.000^†^
	Male	6.6±2.0		6.3±2.0	
Appointment waiting times	< 2 week	6.6±1.9	^∑^ 0.270	7.3±1.8	0.032^‡^
	3-6 week	6.8±2.4		5.6±2.4	
	>7 week	6.8±1.0		5.1±2.2	
BMI^||^	18.5-24.9	7.7±1.5	^∑^ 0.000	5.2±2.6	0.005^‡^
	25-29.9	6.5±2.3		6.0±1.8	
	30-34.9	5.8±1.7		4.4±2.8	
DM^¶^	Yes	5.7±1.8	^ⱡ^ 0.002	4.7±2.5	0.053^†^
	No	6.9±2.1		5.7±2.3	
Antidiabetic medication	Yes	5.7±1.8	^ⱡ^ 0.002	4.7±2.5	^ⱡ^ 0.053^†^
	No	6.9±2.1		5.7±2.3	

Data are presented as ^*^mean±standard deviation; ^†^Mann-Whitney U test; ^‡^Kruskal-Wallis test; ^§^BBPS = Boston Bowel Preparation Scale, ^||^BMI = Body Mass Index, ^¶^DM = Diabetes Mellitus.

The rate of adequate BP (≥5) was significantly lower in those who used Endofalk. The rate of adequate BBPS (≥5) was higher in patients who stated that they used enemas (p = 0.000). Adequate BP (≥5) was higher in those who stated that they used enemas and those who stated that they completely adhered to a clear liquid diet (p = 0.000) (Table 5).

**Table 5:** The effect of preparation administrations on bowel cleansing. Istanbul, Turkey, 2018

Parameters		Bowel preparation adequacy (N=306)		p
		Adequate BBPS^§^ ≥5	Inadequate BBPS^§^ < 5	
		n(%)	n(%)	
BP^||^ regimens	Endofalk	6 (20)	24 (80)	0.000^*^
	Sodium phosphate (NaP)	165 (80.9)	39 (19.1)	
	Sennoside	48 (76.2)	15 (23.8)	
	Polyethylene glycol (PEG)	9 (100)	0 (0)	
Drinking amount of BP^||^ regimen	Never used	0 (0)	3 (100)	0.057^†^
	Used 1/2	9 (75)	3 (25)	
	Used 3/4	18 (75)	6 (25)	
	Used all	201 (75.3)	66 (24.7)	
Enema	Yes	213 (78.9)	57 (21.1)	0.000^‡^
	No	15 (41.7)	21 (58.3)	
Amount of clear fluids	>3 L^¶^	152 (77.2)	45 (22.8)	0.153^*^
	1-3 L^¶^	76 (69.7)	33 (30.3)	
Compliance to restricted clear liquid diet ^#^	Completely	178 (76.7)	54 (23.3)	0.000^†^
	Partially	50 (73.5)	18 (26.5)	
	Never	0 (0)	6 (100)	

Data are presented as ^*^Chi-Square test; ^†^Fisher-Freeman-Halton test; ^‡^Yates Continuity Correction test; ^§^BBPS = Boston Bowel Preparation Scale; ^||^BP = Bowel preparation; ^¶^L = Liter.

Although not included in the tables, other findings obtained from the research can be summarized as follows: The types of BP medications used showed similar distribution between the study and control groups (p = 0.281). In the IG, there were no patients who did not use the preparation medication, and the rate of those who stated that they drank all was higher than the CG [138 (92%) *vs.* 129 (82.7%), p=0.000]. The rate of those who stated that they follow entirely the clear liquid diet in the IG was significantly higher than the CG [120 (80%) *vs.* 112 (71.8), p = 0.025]. In terms of the difficult aspects of preparing for a colonoscopy, patients in IG reported more difficulty in following the clear liquid diet [24(16%) *vs.* 12(7.8%), p=0.044]. Patients in CG, on the other hand, stated that they had more difficulty in drinking the preparation medications [IG: 39 (26%) *vs.* CG: 65 (42.5%), p = 0.003]. Those who stated that they had no difficulty in colonoscopy preparation were significantly higher in the IG [48 (32%) *vs.* 24 (15.7%), p = 0.001].

## Discussion

In this study, we aimed to evaluate the effect of enhanced patient education on BP quality and colonoscopy results. The intervention and control groups participating in the study showed a similar distribution in the baseline of socio-demographic and clinical characteristics. The patients’ mean age is compatible with the age of 50 recommended for the onset of colonoscopy screening^23^ ^-^ ^24^. Our patients’ data, such as age, sex, and BMI, are in line with similar study results^12^ ^-^ ^17^ ^,^ ^25^ ^-^ ^27^. 

Adequate BP rates are one of the main performance criteria for a colonoscopy that enables detecting polyps >5 mm, and means there is no residue, no stool, and/or opaque liquid in colon segments. It is recommended that this rate be ≥90%. Inadequate BP leads to prolonged CIT, decreased ADR, and increased need for a repeat colonoscopy. Inadequate BP rates are reported as 20-25%^2^ ^,^ ^4^ ^-^ ^7^. In the study, BBPS ≥5 was determined as an adequate BP criterion. In the literature, the BBPS cut-off scores used are ≥5, ≥6, or ≥ 6 + each segment score ≥2^5^ ^,^ ^11^ ^-^ ^12^ ^,^ ^15^ ^,^ ^28^ ^-^ ^29^. The completion of the procedure means achieving the CI. The CIR is recommended to be a minimum ≥ of 90%^4^ ^-^ ^5^. In the study, total BBPS mean score, adequate BP rates, and CI rates were found higher than CG in IG. There was no CI failure due to inadequate BP in the IG and the mean score was above the BBPS ≥6 recommended in guidelines for adequate BP. Considering that there is no difference in the education levels between groups, these results obtained from the IG show that the verbally enhanced patient education and reminder phone calls before the procedure make a significant difference in the patients’ BP quality. In the CG, the rate of unsuccessful CI associated with inadequate BP is 17.6%. These results in CG should be evaluated in terms of increases in repeat colonoscopies, workload, and health care costs. Results of a meta-analysis study reveal that bowel adequacy is higher in groups with enhanced education than in the control group^16^. In this meta-analysis, in the eight studies, adequate BP assessment was done using BBPS, and BBPS scores were found to be higher in groups with enhanced patient education than in control groups. In the literature, studies evaluate the effect of training with different tools and methods on adequate BP. Face to face education method was used in two of these studies^11^ ^,^ ^17^. In both studies, it was reported that the total BBPS scores and adequate BP rates in the study groups were higher than in the control groups. In another study, the study group was given reminder phone calls before the procedure, and BBPS scores, adequate BP rates, and CIR were found higher in the study group^12^. A study in which patients were educated via WeChat and SMS revealed that the patients in both study groups had higher BBPS scores than in the control group^14^. In another study using the short message service, BBPS scores and adequate BP rates were higher in the study group^15^. In two studies, pre-procedure educational videos were sent to patients in the study groups, and similar results were obtained^13^ ^,^ ^30^.

The CIR reported by retrospective studies without enhanced patient education is similar to our CG. CIR was 73.4% in one of these studies and 61% in the other^10^ ^,^ ^31^. In another retrospective study, CIR was reported to be 72.1% and 75.4% in patients whose bowel cleansing was evaluated as moderate and poor, respectively^32^. On the other hand, a retrospective study reported a CIR rate of 90%^33^. In our study and most of the studies, CI rates do not reach the 90% suggested by the guidelines. Although not the only cause of CI failure, inadequate bowel cleansing is the most important and modifiable aspect, and it should be considered. 

Polyp detection and polypectomy rates are considered a criterion for ADR. The polyp detection rate is a minor performance criterion that indicates at least one polyp detection for patients >50 years old. The minimum standard is set at 40%^4^ ^-^ ^5^. In our study, we found PDR quite close to the minimum standard in the IG. When studies conducted with groups receiving the enhanced patient education sessions are examined: ADR and PDR rates were statistically significantly higher in study groups in some studies^12^ ^,^ ^15^ ^-^ ^17^ ^,^ ^30^, while in some studies, there was no significant difference^11^ ^,^ ^13^ ^-^ ^14^.

The CIT may vary in each session and usually takes 10-20 minutes. Inadequate BP is a significant predictor of prolonged CIT (≥20 min)^8^. In our study, the mean of CIT was 8.8 minutes, and there was no difference between groups. When we evaluate this parameter in the studies conducted with groups receiving enhanced patient education, the duration of the study groups was shorter in three studies^12^ ^-^ ^14^ ^,^ ^16^. There was no difference between the groups in the two studies, similar to our results^11^ ^,^ ^30^.

Patient-related demographic and clinical characteristics may affect adequate BP. A systematic review’s results show that >65 age, male sex, high BMI, diabetes mellitus, and constipation are associated with inadequate BP^34^. There was no difference between the BBPS scores in terms of age and sex in our IG. We interpret this result as enhanced patient education in the IG may have eliminated age and sex differences. In our CG, unlike similar studies^13^ ^,^ ^35^, we found lower BBPS scores in women. In our IG, the waiting times for colonoscopy did not affect BBPS scores. In the CG, however, as the waiting time extended, BBPS scores and adequate BP rates decreased gradually. Two studies support this result^36^ ^-^ ^37^. It may not be feasible for each unit to reduce waiting times for colonoscopy due to patient load. For these reasons, we think that reminder phone calls before the procedure will positively affect adequate BP and CI rates. 

In our study, BBPS scores were higher in patients with an average weight in both groups, and results suggest that diabetes mellitus may negatively affect BBPS scores as in similar studies^13^ ^,^ ^35^. In the study, the BP adequacy was found to be the highest in the polyethylene glycol (PEG) group and the lowest in the Endofalk group. Similar to our results, PEG was found in one study^33^ to be more effective than sennoside and sodium phosphate in terms of adequate BP rate. In contrast, in another study^38^, no difference was found between PEG and sennoside. Our study results indicate that enema administration and full compliance with the clear liquid diet before the procedure have an essential effect on bowel cleansing.

Being a single-center study and the lack of comparison in terms of workload and costs constitutes the main limitations of the study.

## Conclusion

This study provides strong evidence that the nurse-performed enhanced patient educations via face-to-face training and reminder calls have a significant effect on adequate BP and CI rates. We believe that the enhanced education will provide patients with a better understanding of the BP process and increase their compliance. Besides, these communication-based education sessions will increase the collaboration between patients on the one hand and nurses and doctors on the other. The enhanced patient education, by creating a domino effect, can increase adequate BP, CI, and AD rates, thus decreasing repeated colonoscopies. Hence, the perioperative workload of colonoscopy nurses will be reduced. It will also provide additional benefits such as reduced costs in the healthcare system. Therefore, it is suggested that patient education in gastrointestinal endoscopy units should be designed according to the patient’s socio-demographic and clinical characteristics and provided by health care professionals. Education sessions should also be supported by different tools and methods such as illustrated brochures, videos, education groups, phone calls, short messages, social media applications, and smartphone technology.
